# Comparison of HemoCue^® ^hemoglobin-meter and automated hematology analyzer in measurement of hemoglobin levels in pregnant women at Khartoum hospital, Sudan

**DOI:** 10.1186/1746-1596-7-30

**Published:** 2012-03-21

**Authors:** Ishag Adam, Samah Ahmed, Mahmoud H Mahmoud, Mohammed I Yassin

**Affiliations:** 1 Faculty of Medicine, University of Khartoum, P.O. Box 102, Khartoum, Sudan

## Abstract

**Background:**

Assessment of hemoglobin is one of the most reliable indicators for anemia, and is widely used to screen for anemia among pregnant women. The HemoCue^® ^has been widely used for as a point-of-care device for hemoglobin estimation in health facilities. Previous studies showed contradictory results regarding the accuracy of HemoCue^®^.

**Methods:**

This was a hospital-based cross sectional study carried- out among pregnant women at Khartoum hospital in Sudan to find out whether the measurement of hemoglobin concentration by HemoCue^® ^using venous or capillary samples was comparable to that of the automated hematology analyzer as standard. Bland and Altman method was used to compare the measurements with an acceptable difference of ± 1.0 g/dl.

**Results:**

Among the 108 subjects in this study the mean (SD) level of hemoglobin level using HemoCue^® ^venous sample, HemoCue^® ^capillary sample and automated hematology analyzer were 12.70 (1.77), 12.87 (2.04) and 11.53 (1.63) g/dl, respectively. Although the correlations between the measurements were all significant there was no agreement between HemoCue^® ^and automated hematology analyzer. The bias + SD (limits of agreement) for HemoCue^® ^venous versus hematology analyzer was 1.17 ± 1.57 (-1.97, 4.31) g/dl, HemoCue^® ^capillary versus hematology analyzer was 1.34 ± 1.85 (-2.36, 5.04) g/dl, and HemoCue^® ^venous versus HemoCue^® ^capillary samples was 017 ± 1.90 and (3.97-3.63) g/dl.

**Conclusion:**

Hemoglobin concentration assessment by HemoCue^® ^using either venous or capillary blood samples has shown unacceptable agreement with automated hematology analyzer.

**Virtual slides:**

The virtual slide(s) for this article can be found here: http://www.diagnosticpathology.diagnomx.eu/vs/8797022296725036

## Introduction

Anemia is one of the most important causes of morbidity and mortality in developing countries, especially among pregnant women [1]. The world prevalence of anemia in pregnant women and non-pregnant women is 41.8% and 30.2% respectively [2]. Pregnant Sudanese women are susceptible to anemia regardless to their age and parity and anemia is one of the leading causes of maternal and perinatal morbidity and mortality [3-5].

Assessment of hemoglobin is one of the most reliable indicators for anemia, and is widely used to screen for anemic individuals, and to evaluate responses to interventions [6]. Hemoglobin concentration is routinely measured using automated hematology analyzers. Although, these are very accurate and reliable, they are expensive and problems of samples transport to the laboratory may delay treatment resulting in preventable deaths [7]. In poor resources where automated hematology analyzer is not available other methods of low price and that require less skill are highly needed. Although Cyanmethemoglobin method is cheaper and often used, it takes more time. The semi-quantitative gravimetric copper sulfate method which is used in blood donation is very easy and inexpensive; it does not provide an acceptable degree of accuracy.

The HemoCue^® ^hemoglobin photometer has been widely used for as a point-of-care device for hemoglobin estimation in mobile blood donations and critical care areas in health facilities [8]. Previous studies showed contradictory results regarding the accuracy of HemoCue^®^; some of research reported a high accuracy of HemoCue^® ^compared with standard laboratory methods [9,10]. However, others did not recommend this device in general practice [11-13]. Few published data exist on the accuracy of HemoCue^® ^in the measurement of hemoglobin during pregnancy [14,15]. The aim of this study was to compare the accuracy of HemoCue^® ^using venous and capillary samples with that of the automated hematology analyzer in the measurement of hemoglobin among pregnant Sudanese women at Khartoum Hospital.

## Material and methods

This was a cross sectional study carried- out among pregnant women at Khartoum hospital in Sudan during the period of October through December 2011. Convenient sampling method was used in the study in which all available subjects who fulfilled the inclusion criteria at the Khartoum hospital antenatal care clinic were recruited in the study every day until total number of sample size was achieved. Sample size of 108 subjects was calculated based on a 2-sided hypothesis tests using Epiinfo with 80% power and confidence interval of 95%. After signing an informed consent venous and capillary blood samples were taken from each woman to be measured by HemoCue^® ^(venous and capillary) and the automated analyzer.

Capillary blood samples were collected by finger prick in the middle finger of left hand, after cleaning and massaging the finger to facilitate blood flow. Venous blood samples were collected into vacutainer tubes and were analyzed immediately by HemoCue^® ^and the tubes were sent to the medical laboratory of the hospital for the analyses by automated analyzer.

### HemoCue^® ^portable photometer

The HemoCue^® ^B-Hemoglobin system (HemoCue^® ^AB, Ängelholm, Sweden) consists of disposable microcuvettes containing reagent in a dry form and a single purpose designed photometer. The microcuvettes were stored in a dry place at room temperature. Once opened, they were tightly closed and stored at the same conditions to maintain their integrity and shelf life. The reaction in the microcuvette is a modified azide-methemoglobin reaction. Sodium deoxycholate haemolyses erythrocytes and hemoglobin is released. Sodium nitrite converts hemoglobin to methemoglobin which, together with sodium azide, gives azidemethemoglobin. The absorbance is measured at two wavelengths (570 nm and 880 nm) in order to compensate for turbidity in the sample. The test was performed as stated by the manufacturer [16].

### Automated hematology analyzer

The Sysmex KX21N (Sysmex Corporation, Kobe, Japan) is an automated blood cell counter intended for in vitro diagnostic use in clinical laboratories. It is a compact, fully automated hematology analyzer with simultaneous analysis of 18 parameters in whole blood mode and capillary blood mode. It measures the hemoglobin concentration using a non-cyanide hemoglobin method (STROMATOLYSER WH). The instrument has been proven to provide accurate and reliable results including hemoglobin concentrations [17,18]. The test was performed as stated in the manufacturer's manual [19].

### Quality control

The HemoCue^® ^photometer was checked on a daily basis using the control cuvette and a standard of known concentration. A three set controls were run daily to ensure the function of the Sysmex.

### Statistics

All statistical analyses were performed using SPSS software (SPSS Inc., Chicago, IL, USA, version 16.0). Hemoglobin levels were measured using two instruments; HemoCue^® ^and automated hematology analyzer. HemoCue^® ^used two types of samples; venous as well as capillary samples. Pairs of hemoglobin measurements were compared as follows: HemoCue^® ^venous sample versus automated hematology analyzer, HemoCue^® ^capillary sample versus automated hematology analyzer, HemoCue^® ^venous sample versus HemoCue^® ^capillary sample. Pearson Correlation analysis was performed and correlation coefficient (r) was calculated. The Mean of differences (bias), standard deviation of differences (SD), and limits of agreement (Mean ± 2 × SD) were calculated according to the Bland and Altman method [20]. Limits of agreement not exceeding ±1 g/dl between any two pairs of methods were considered to be clinically acceptable.

### Ethics

This study was completely conducted voluntarily and all respondents were given brief explanations about the purpose and procedures of the study to be used. Voluntarily consent was signed by each subject before taking the blood. Ethical clearance was obtained from the institute Board.

## Results

A total number of 108 pregnant women were recruited in this study. Their mean (SD) of the age and gestational age were 27.6 (6.8) years and 24.9 (10.2) weeks.

The mean (SD) hemoglobin level was 12.70 (1.77), 12.87 (2.04) and 11.53 (1.63) g/dl using HemoCue^® ^venous sample, HemoCue^® ^capillary sample and automated hematology analyzer, respectively (Table 1).

**Table 1:** Hemoglobin level measured using HemoCue^® ^venous sample, HemoCue^® ^capillary sample, and automated hematology analyzer (g/dl)

Method of measurement	Mean ± SD	Median	(min; max)
HemoCue^® ^(venous)	12.70 ± 1.77	12.80	(8.90; 17.90)
HemoCue^® ^(capillary)	12.87 ± 2.04	12.90	(8.20; 17.70)
Hematology analyzer	11.53 ± 1.63	11.40	(8.10; 15.00)

### HemoCue^® ^venous versus automated hematology analyzer

**Figure 1:**
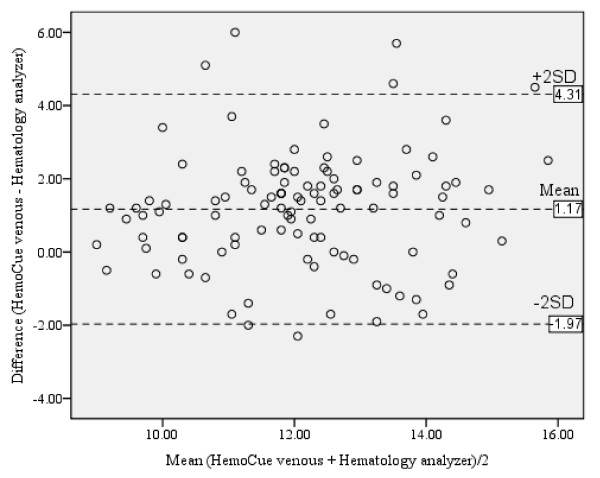
**Bland and Altman plot for hemoglobin level by using HemoCue^® ^venous sample versus automated hematology analyzer (g/dl)**.

There was a positive correlation (*r *= 0.58, *P *< 0.001) between hemoglobin levels by using HemoCue^® ^venous sample versus automated hematology analyzer. The mean difference with limits of agreement between the two reading was 1.17 (-1.97, 4.31) g/dl (Table 2 and Figure 1).

**Table 2:** Correlation, bias, and limits of agreement between hemoglobin level using HemoCue^® ^and automated hematology analyzer

Comparison of methods	Correlation Coefficient	Bias ± SD (95%CI)	Limits of agreement
HemoCue^® ^(venous) vs. Hematology analyzer	0.58	1.17 ± 1.57 (0.87; 1.47)	-1.97 to 4.31
HemoCue^® ^(capillary) vs. Hematology analyzer	0.51	1.34 ± 1.85 (0.99; 1.69)	-2.36 to 5.04
HemoCue^® ^(venous) vs. HemoCue^® ^(capillary)	0.51	-0.17 ± 1.90 (-0.53; 0.19)	-3.97 to 3.63

### HemoCue^® ^capillary versus automated hematology analyzer

**Figure 2:**
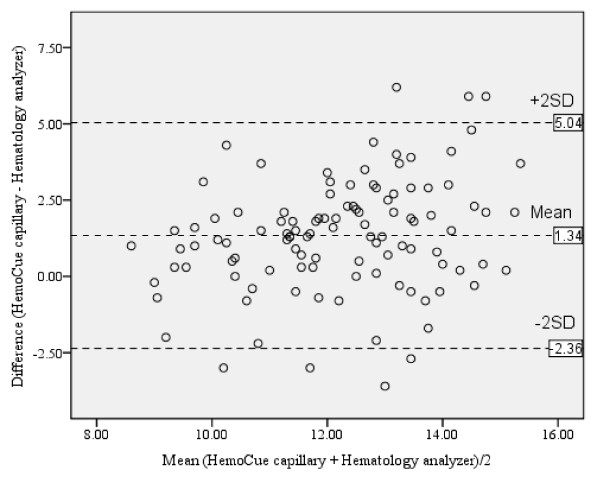
**Bland and Altman plot for hemoglobin level by using HemoCue^® ^capillary sample versus automated hematology analyzer (g/dl)**.

There was a positive correlation between hemoglobin levels by using HemoCue^® ^capillary sample versus automated hematology analyzer (*r *= 0.51, *P *< 0.001). The mean difference with limits of agreement between hemoglobin levels by using HemoCue^® ^capillary sample versus automated hematology analyzer was 1.34 (-2.36, 5.04) g/dl (Table 2 and Figure 2).

### HemoCue^® ^venous versus HemoCue^® ^capillary sample

**Figure 3:**
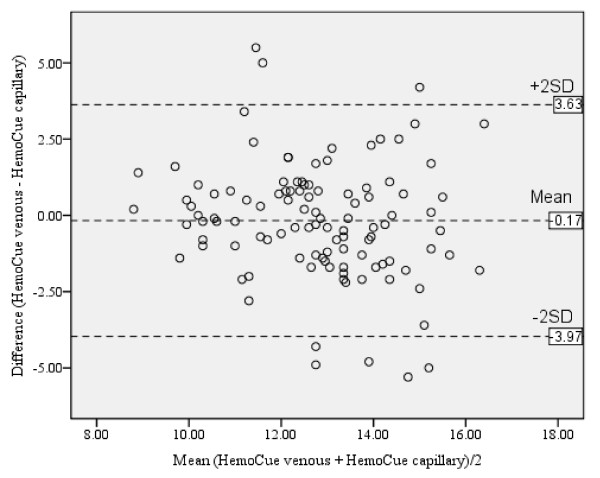
**Bland and Altman plot for hemoglobin level by using HemoCue^® ^venous sample versus HemoCue^® ^capillary sample (g/dl)**.

There were positive correlation between hemoglobin levels by using HemoCue^® ^venous sample versus HemoCue^® ^capillary sample (*r *= 0.51, *P *< 0.001). The mean difference with limits of agreement between hemoglobin levels by using HemoCue^® ^venous sample versus HemoCue^® ^capillary sample was -0.17 (-3.97, 3.63) g/dl (Table 2 and Figure 3).

According to the previously pre-defined clinical acceptable limits of ± 1 g/dl, the 2 methods could not be considered as interchangeable.

## Discussion

Recently HemoCue^® ^portable hemoglobin photometer using venous or capillary blood samples has been widely used for quick assessment of hemoglobin concentrations; especially in poor settings where skills and resources are limited.

The accuracy of HemoCue^® ^for measuring hemoglobin in clinical settings is still a matter of controversy. The results of this study have shown that the hemoglobin concentration of HemoCue^® ^using either venous or capillary blood samples have lower level of precision and was not comparable with that of automated hematology analyzer; the limits of agreement were larger than the predefined clinically acceptable limits of ±1 g/dl. This is goes with the previous findings of the studies conducted among pregnant women at high altitude [14] and adult patients hospitalized in surgical intensive care unit [21]. The difference between the readings has been explained by the use of only one microcuvette with HemoCue^® ^especially when using capillary blood samples and laboratory values, and advised loading multiple microcuvettes and averaging the hemoglobin values obtained has been proposed [22].

However, other studies showed that the results obtained by using HemoCue^® ^for hemoglobin assessment among pregnant women were comparable to that of automated hematology analyzer as a standard [9]. Bernard *et al.*, found that the results of hemoglobin concentration among pregnant and non-pregnant populations using HemoCue^® ^were comparable to that of automated hematology analyzer and Cyanmethemoglobin methods [23]. Other studies which were conducted in different settings and populations such as patients with gastrointestinal bleeding, surgical patients repeated measurement of one sample, urban general practice, neonates, patients undergoing aortic surgery in the theatre and blood donors recommended HemoCue^® ^for the hemoglobin estimation [8,24-31]. Paiva et al. found that HemoCue^® ^was more appropriate for capillary compared to venous blood samples [32]. However, there was within-subject variability of capillary blood hemoglobin values that might explain the unreliability of the method, and it has been shown that two capillary samples taken from different fingers of the same subjects had hemoglobin concentrations differing by/ more than two g/dL using the HemoCue^® ^[6].

In spite of the non-acceptable agreement of HemoCue^® ^with automated hematology analyzer in this study, the HemoCue^® ^is however simple to use, need minimum training, cheap, and gives an immediate result. Furthermore, it is useful in clinical and epidemiological settings where finger puncture allows capillary blood sampling as an easy technique which is less resource-intensive than vein puncture, and is more acceptable to patients and the community.

## Conclusion

Hemoglobin concentration assessment by HemoCue^® ^using either venous or capillary blood samples does not have an acceptable agreement with automated hematology analyzer. Therefore this study does not recommend this device for hemoglobin concentration assessment among pregnant women.

## Competing interests

The authors declare that they have no competing interests.

## Authors' contributions

IA and MIY designed the study and participated in the statistical analyses, SA and MH conducted the clinical work. MH conducted the lab work. All the authors approved the draft and the final paper.
